# Probability of hospitalisation and death among COVID-19 patients with comorbidity during outbreaks occurring in Mexico City

**DOI:** 10.7189/jogh.12.05038

**Published:** 2022-11-08

**Authors:** José Sifuentes-Osornio, Ofelia Angulo-Guerrero, Guillermo De Anda-Jáuregui, Juan L Díaz-De-León-Santiago, Enrique Hernández-Lemus, Héctor Benítez-Pérez, Luis A Herrera, Oliva López-Arellano, Arturo Revuelta-Herrera, Ana R Rosales-Tapia, Manuel Suárez-Lastra, David Kershenobich, Rosaura Ruiz-Gutiérrez

**Affiliations:** 1 Departamento de Medicina, Instituto Nacional de Ciencias Médicas y Nutrición Salvador Zubirán, Mexico City, Mexico; 2 Secretaría de Educación, Ciencia, Tecnología e Innovación, Gobierno de la Ciudad de México, Mexico City, Mexico; 3 Computational Genomics Division, Instituto Nacional de Medicina Genómica, Mexico City, Mexico; 4 Centro de Ciencias de la Complejidad, Universidad Nacional Autónoma de México, Mexico City, Mexico; 5 Dirección General de Cómputo y de Tecnologías de Información y Comunicación, Instituto de Investigaciones en Matemáticas Aplicadas y en Sistemas, Universidad Nacional Autónoma de México, Mexico City, Mexico; 6 Instituto de Investigaciones Biomédicas, Universidad Nacional Autónoma de México, Mexico City, Mexico; 7 Instituto de Geografía, Universidad Nacional Autónoma de México, Mexico City, Mexico; 8 Facultad de Ciencias, Universidad Nacional Autónoma de México, Mexico City, Mexico

## Abstract

**Background:**

We compared the probability of hospitalization and death caused by COVID-19 in patients with comorbidities during three periods defined for this study: first-wave (FW), interwave period (IP), and second-wave (SW) observed in Mexico City.

**Methods:**

In this registry-based study, we included individuals over 20 years of age. During the FW (symptomatic), the IP, and the SW (symptomatic and asymptomatic), participants were diagnosed using nasopharyngeal swabs. Symptomatic individuals with risk factors for serious disease or death were referred to the hospital. SARS-CoV-2 infection was defined by RT-qPCR in all hospitalized patients. All data were added to the SISVER database. Bayesian analysis and False Discovery Rate were used for further evaluation.

**Results:**

The study included 2 260 156 persons (mean age of 43.1 years). Of these, 8.6% suffered from DM, 11.6% arterial hypertension, and 9.7% obesity. Of the total, 666 694 persons tested positive (29.5%). Of the infected persons, a total of 85 587 (12.8%) were hospitalized: 24 023 in the FW; 16 935 in the IP, and 44 629 in the SW. Of the hospitalized patients, there were 42 979 deaths (50.2%), in the FW, 11 964 (49.8%), in the IP, 6794 (40.1%), and in the SW 24 221 (54.3%). The probability of death among individuals hospitalized with or without comorbidities increased consistently in all age groups. A significant increase in the Fatality Rate was observed in individuals with comorbidities (1.36E-19< = FDR< = 3.36E-2). A similar trend was also observed in individuals without comorbidities (1.03E-44< = FDR< = 5.58E-4).

**Conclusions:**

The data from this study show a considerable increase in the number of detected cases of infection between the FW and SW. In addition, 12.8% of those infected were hospitalized for severe COVID-19. A high mortality rate was observed among hospitalized patients (>50%). An age-dependent probability of death was observed with a positive trend in hospitalized patients with and without comorbidities.

The original outbreak of SARS-CoV-2 virus (COVID-19) occurred in the province of Wuhan, China, in November 2019 [1]. It then spread rapidly worldwide and was categorized as a pandemic by the World Health Organization (WHO) in 2020 [2]. During the progress of the pandemic in several regions of the world, it was observed that being elderly, having chronic obstructive pulmonary disease, diabetes mellitus (DM), arterial hypertension (AHP), or obesity were common features among individuals who required hospitalization or died and were associated with increased risk for the development of the disease [3] caused by the COVID-19.

This association has been systematically observed throughout the world, the basis of which seems to be the pro-inflammatory state suffered by patients living with these chronic non-communicable diseases [4]. It has also been observed that patients with uncontrolled AHP develop an excess of receptors ACE-2 [5]. Since these conditions are common in adults around the world, obesity and being overweight particularly, as well as uncontrolled or undiagnosed DM or AHP, COVID-19 has found fertile ground in which to take root and spread. The disease has a good chance of permanently residing in this environment and infecting other individuals with poor or limited immunity mechanisms, such as people with organ transplants, patients with neoplastic diseases undergoing chemotherapy, patients with chronic rheumatic diseases under immunosuppressive treatment, among other groups [6].

The appearance of COVID-19 in the previously healthy population has attracted much attention recently since this disease has been observed in children, adolescents, and young adults who are at risk of death or serious deterioration, in addition to the risks of kidney, lung, or liver complications, which can lead to permanent disability. It has also been observed that the risk of complications increases with the number of pre-existing comorbidities. Complications tend to be more frequent in men than in women, and in those patients who were admitted to intensive care units or required mechanical ventilation [7]. The pre-existence of overweight/obesity, DM, or AHP [8] has meant that these complications have been observed frequently in the Mexican population. The prevalence of DM has been reported in adults over 20 years of age at rates of 10.3% throughout the country and 12.7% in Mexico City (CDMX); in the case of AHP, 18.4% and 20.2%, respectively. While in the case of obesity, the figures are 75.2% (39.1% overweight and 36.1% obesity) and 75.9% (overweight 40.6% and obesity 35.3%), for the country as a whole and CDMX, respectively [8].

The CDMX Government has implemented various precautionary measures in the population based on the recommendations of the WHO, the Federal Government, and the Metropolitan Committee on Health, [9-11]. In summary, these were: 1) application of an epidemiological mobility/risk of transmission indicator; 2) suspension of classes, commercial activities, and all kinds of face-to-face activities in the offices and dependencies of the CDMX government; 3) promotion of the use of face masks while in public places and frequent handwashing with soap and water or at minimum with gel alcohol; 4) provision of a medical guidance service for people presenting with symptoms of respiratory illness, including the instruction to stay at home on the CDMX website and via Facebook and periodic monitoring; 5) detection and follow-up of patient contacts; 6) opening of several acute care hospitals for patients with COVID-19; 7) opening of the first stage of the Temporary Unit to care for patients with COVID-19, and Oxygen support at home, both programs sustained by private donations.

In prior studies carried out by other Mexican groups [12,13] and in a study carried out by this group in the CDMX (our unpublished observations), a strong association between the severity of COVID-19, being over 40 years of age, and certain non-communicable chronic diseases, in particular DM, high blood pressure and obesity, was observed. This group has previously analyzed the impact of comorbidities and different age ranges on the risk of developing complications. However, the evolution of the pandemic suggests the possibility that the changes could have occurred because of the interventions, as well as being due to changes in population dynamics, for example, mobility or the emergence of variants of concern in the country, among other conditions [14,15]. Therefore, the objective of this study was to compare the probability of death caused by COVID-19 in patients with comorbidities during three periods of the pandemic that we defined as first-wave (FW), the epidemiological weeks 13-28, 2020, March 23 to July 12, 2020), interwave period (IP), the epidemiological weeks 29-43 of 2020, July 13 to October 25, 2020), and second-wave (SW), the epidemiological weeks 44/2020 – 13/2021, October 26, 2020, to March 29, 2021), based on the different case fatality rates (CFR) that we observed in the CDMX during the study period.

## METHODS

### Study population and design

This registry-based study included individuals aged over 20: 193 579 people tested during the FW; 338 639 during the IP, and 1 727 938 during the SW. All the subjects included were evaluated at the outpatient clinics, emergency services, strategically located health kiosks, and hospitalization centers of the health institutions of the CDMX between March 23, 2020, and March 29, 2021. All the people included during the first wave presented with one or more of the following symptoms: fever, cough, sore throat, dyspnoea, rhinorrhoea, nasal congestion, conjunctivitis, myalgia, arthralgia, headache, for more than one day. The people included during the interwave period, and the second wave included both symptomatic and asymptomatic individuals whose samples were taken in specially established kiosks in different locations in CDMX. The study was approved by the Research and Research Ethics Committees of the Instituto Nacional de Ciencias Médicas y Nutrición Salvador Zubirán (IRB: 3347).

In each case, information was obtained on demographic data, recent trips (during the FW), contact with other suspected or confirmed cases, comorbidities, signs, and symptoms, and monitoring of the evolution of their illness, using a COVID-19 epidemiological case study format prepared by the Ministry of Health (data on the dates of hospitalization and death were included as an outcome). Symptomatic individuals who tested positive on the diagnostic test and having one or more risk factors for serious disease or death (age ≥65 years, AHP, DM, obesity, chronic obstructive pulmonary diseases (COPD), ischemic heart disease, cardiovascular disease (CVD), temperature ≥39°C, respiratory rate ≥24 rpm, saturation O2 < 92%, dyspnoea, abdominal pain) were referred to the reference institutions participating in the program for a thorough evaluation and better care.

### Definition of waves and interwave period

In Mexico, the first identified case of COVID-19 occurred on February 27, 2020, and by March 18, the first death from that cause was recorded. Five days later, on March 23, 2020, Mexico announced the start of the quarantine, and the decision was made to close schools and suspend nonessential activities. A week later, on April 2, 2020, a state of emergency was declared throughout the country. During this initial phase of the pandemic, Mexico ranked third in the world in the number of infections and deaths recorded, behind the USA and Brazil.

To avoid using a biased definition of the study periods, we defined a wave as the period between two local minima in the time series of epidemiologic parameters (that is, the second derivative of the time series is zero). To avoid biases from changes in testing protocols, we used the time series of weekly new confirmed hospitalized cases and weekly deaths in hospitals. We then defined the interwave as the period between the end of a wave and the start of another.

### Diagnostic procedures

SARS-CoV-2 infection was detected by real-time reverse-polymerase chain reaction (RT-qPCR) with reverse transcriptase by Applied Biosystems 7500 (Applied Biosystems, Foster City, CA, USA), exclusively, between March 23 and November 30, 2020, as previously described [16]. Subsequently, the SARS-CoV-2 Roche SARS-CoV-2 rapid antigen test (Roche, Basel, Switzerland) or Abbott BinaxNOWTM 88 COVID-19 Ag Card (Abbott Laboratories, Abbott Park, IL, USA) determination was used for antigen detection in the nasopharyngeal swab following the manufacturer’s recommendations, between December 1, 2020, and March 29, 2021. In all hospitalized patients, the diagnosis of COVID-19 was confirmed by RT-qPCR.

All data from hospitalized patients and outpatients residing in the CDMX were added to the Epidemiological Surveillance System for Respiratory Diseases (SISVER) database [17] for each medical unit and included demographic data, medical unit, results of the SARS CoV-2 diagnostic test, residence, age, sex, date of onset of symptoms, date of admission to hospital, comorbidities, and date of death, among others. Additionally, the CDMX Government’s Digital Agency for Public Innovation completed the data, incorporating detailed information on the cases, as well as other indicators such as the number of beds available for COVID-19 care, hospitalization rates, and overall availability of beds.

### Statistical analysis

Considering that, in the context of COVID-19, the presence of certain comorbidities has been associated with more unfavorable outcomes, such as hospitalization, intubation, and death, a classification model was implemented that identifies individual patients’ risk to either outcome, based on their associated comorbidities. For that, the use of conditional probabilities over descriptive statistics was chosen, since the former are usually less susceptible to sampling errors and normalize the results to the conditions given by the independent variables of interest. This allows us to answer questions that are of interest to the individual. So, if the fact of interest is the likelihood of death when presenting with DM and suffering from COVID-19, there is a conditional probability for obtaining the answer to that specific question for both the event of interest (dependent variable) and for the chosen precondition (independent variable). Then, the conditional probability of event A given event B is obtained as follows: P(A\B) = P(A∩B)/P(B), and when we are interested in multiple events or conditions, the conditional probability is obtained as follows: P(A\B1⋀B2⋀... ⋀Bn) = P(A∩B1B2∩... ∩Bn)/P(B1B2∩... ∩Bn) [18].

In this study, analysis for each 20-year age bracket was generated for each period: the FW, the IP, and the SW. In each age bracket, a Bayesian analysis was performed on the three comorbidities: DM, AHP, and obesity [19]. Although the SISVER database may have some “measurement uncertainty”, the Bayesian model is not affected by this kind of “uncertainty” since the potential biases are incorporated into the priors from the start. Thus, the resulting analysis model was “strengthened” by using this strategy.

For all cases, the following conditional probabilities were calculated: a) the probability of death given that the person was positive for SARS-CoV-2 infection and had the comorbidity, and b) the probability of death given that the person developed COVID-19, was hospitalized, and had the comorbidity. Furthermore, the same conditional probabilities were obtained for patients without any of the following comorbidities: DM, AHP, obesity, COPD, cardiac diseases, or renal failure. These patients were considered as the healthy population baseline for this study. Finally, the conditional probabilities for each time frame were compared to identify what had changed in time for every case. It is important to point out that all the conditional probabilities share a base condition, namely the SARS-CoV-2 testing that was performed on many individuals who voluntarily attended the kiosks due to the recent appearance of symptoms or their suspicion of having an asymptomatic infection. The attributable and relative risks for each comorbidity were calculated based on the conditional probability of each comorbidity contrasted against the conditional probability of not having any comorbidity. Relative risks were calculated as odds ratios and attributable risks are shown as differences.

Regarding the sensitivity of the Bayesian analysis, the precision in the results is inversely proportional to the frequency of the conditional event. For instance, if the conditional event B has a frequency of 1000, it can be expected to have a maximum uncertainty of 0.1% peak to peak, or a mean uncertainty of plus or minus 0.0005, in all the probabilities conditioned to B [19].

### Comparative analysis of lethality in hospitalized patients during the waves in contrast to the interwave period

We used Fisher exact test to evaluate whether the proportion of survivors and deaths among hospitalized patients showed differences between the periods evaluated. Since the ratio between survivors and deaths is proportional to the CFR, we concluded that the CFR among hospitalized patients changed during the periods of greater transmission and greater demand for hospitalization.

The following contrasts were made to assess the significance of the prior changes: 1) comparison between hospitalized patients during either of the two waves and in the interwave period and 2) comparison between hospitalized patients in the first peak and the second peak. The comparison was made using Fisher exact test, considering a contingency table (see Table S1 in the **Online Supplementary Document**).

The *P*-values of the multiple comparisons were corrected using the False Discovery Rate (FDR), and an FDR value <0.05 was considered significant.

To avoid Simpson Paradox issues that might obscure differences, these comparisons were performed in subpopulations defined by age group and the combinations of the three comorbidities studied; additionally, the subpopulation of patients not presenting with any of the three specified comorbidities but with any other recorded comorbidity was studied separately from the subpopulation with no recorded comorbidity. We additionally, compared the interwave period with the two waves to gain a better notion of changes in lethality associated with periods of increased transmission and associated with greater demand for hospitalization. Thus, one of our objectives was to evaluate whether there were changes in lethality between the interwave period and the second wave. The rationale was that while in the first wave knowledge of the disease was scarce and detection programs were being developed, by the interwave period, several interventions had been adopted, including increased testing programmes. Given that lethality increases during periods of higher transmission and demand for hospitalization, we calculated the Pearson correlation between weekly CFR for hospitalized patients and hospital occupancy as reported [20].

### Correlation between weekly hospitalized CFR and hospital capacity

We calculated the Pearson correlation between weekly CFR for hospitalized patients in each age group vs hospital capacity (as recorded in the federal dashboard) [20].

## RESULTS

### Descriptive analysis

During the study period, 2 260 156 subjects were included, all having been tested for SARS-CoV-2 infection performed by nasopharyngeal RTq-PCR or antigen testing. The mean age of the subjects was 43.1 years (SD 15.0), 1 172 907 (51.9%) being women. We included 194 647 individuals suffering from DM (8.6%); 262 652 had AHP (11.6%), and 218 769 were obese (9.7%). Of the total of studied subjects, 666 694 tested positive (29.5%) to SARS CoV-2, having a mean age of 45.0 (SD = 15.6). Of those testing positive, 340 811 (51.1%) were women. There was a considerable increase in the number of cases (+472%) of infection in all age groups between the FW and the SW (**Table 1**). The number of subjects in each age bracket was as follows: 20 to 39 years of age: 273 715 (41% of whom tested positive), 140 248 being women (51.1%); 40 to 59 years of age: 269 285 (40.4% of whom tested positive), 139 241 being women (51.7%); 60 to 79 years of age: 109 467 (16.4% of whom tested positive) 53 964 being women (49.3%); 80 years of age and over: 14 227 (2.1% of whom tested positive), 7358 being women (51.7%).

**Table 1:** Sample size of the population tested and infected by SARS-CoV-2 by age groups and COVID-19 waves

Age (years)	Population tested (n)			Population infected (n)		
	**1^st^ wave**	**Interwave period**	**2^nd^ wave**	**1^st^ wave**	**Interwave period**	**2^nd^ wave**
20-39	77 688	150 732	801 731	28 779 (37.0%)*	45 335 (30.0%)	199 601 (24.9%)
40-59	81 752	134 459	678 177	35 420 (43.3%)	45 642 (33.9%)	188 223 (27.8%)
60-79	29 622	47 742	224 981	15 925 (53.8%)	18 662 (39.0%)	74 880 (33.3%)
80	4517	5706	23 049	2365 (52.4%)	2476 (43.4%)	9386 (40.7%)
Total	193 579	338 639	1 727 938	82 489 (42.6%)	112 115 (33.1%)	472 090 (27.3%)

*The percentages show the proportion of infected subjects divided by the total number of subjects tested in each age group and period.

Of the total of infected individuals, 85 587 (12.8%) were hospitalized after being diagnosed as having a severe respiratory acute infection. This total breaks down as follows: 29.1% during the FW; 15.1% during the IP, and 9.5% during the SW.

**Figure 1:**
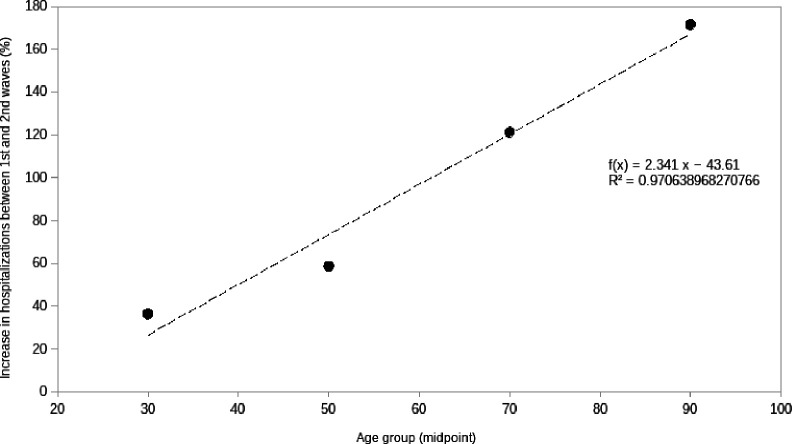
An increase in the number of hospitalizations was observed with age group.

Of the hospitalized subjects, there was an increase of +85.9% between waves, of +36.2% in the case of the group aged 20 to 39 years, +58.6% for the group aged 40 to 59, +121.2% for the group aged 60 to 79 years, and +171.4% for the 80 years or older group (**Table 2**). This shows that the relative increase in hospitalizations between the FW and the SW increased linearly as a function of age groups, as seen in **Figure 1**.

**Table 2:** Sample size of hospitalized patients and mortality by COVID-19 by age groups and pandemic waves

Age (years)	Hospitalizations (n) due to COVID-19			Mortality (n) due to COVID-19		
	**1^st^ wave**	**Interwave**	**2^nd^ wave**	**1^st^ wave**	**Interwave**	**2^nd^ wave**
20-39	3261	2027	4442	716 (22.0%)*	269 (13.3%)	1100 (24.9%)
40-59	10 397	6509	16 485	4350 (41.8%)	1936 (29.7%)	7469 (45.3%)
60-79	8821	6964	19 512	5719 (64.8%)	3619 (52.0%)	12 571 (64.4%)
80	1544	1435	4190	1179 (76.4%)	970 (67.6%)	3081 (73.5%)
Total	24 023	16 935	44 629	11 964 (49.8%)	6794 (40.1%)	24 221 (54.3%)

*The percentages show the proportion of deaths divided by the total number of subjects hospitalized in each age group and period.

### Comparative analysis of the lethality in hospitalized patients during the waves in contrast with the interwave period

**Figure 2:**
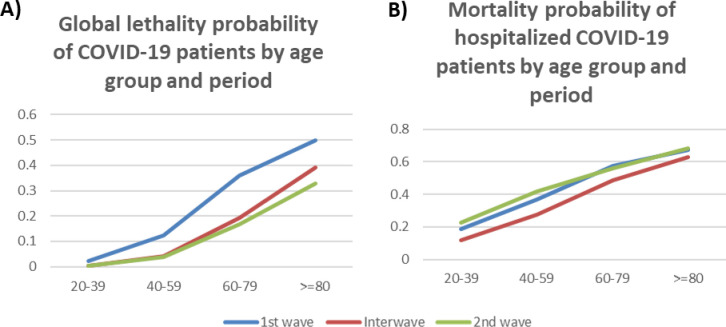
A) Global lethality probability of COVID-19 patients by age group and period and B) Mortality probability of hospitalized COVID-19 patients by age group and period using Bayes probability test.

During the study, there were 42 979 deaths among the 85 587 hospitalized individuals (50.2%), being 11 964 in the FW, 6794 in the IP, and 24 221 in the SW, an increase of +102.4% between the two waves. By age, the percentage increases were: +53.6% in the case of the group aged 20 to 39, +71.7% for the group aged 40 to 59, +119.8% for the group aged 60 to 79, and +161.3% for the group aged 80 and older (**Table 2**). Given that both the numbers of tests performed, and subjects tested were notably greater during the IP and the SW (**Table 1**), the probability of death was significantly smaller in all age groups of the general population, as shown in **Figure 2** and Table S2 in the **Online Supplementary Document**. In contrast, the probability of death among individuals hospitalized for COVID-19 with or without comorbidities increased systematically in all age groups, except for the group aged 60 to 79, which remained constantly high (**Figure 2** and Table S3 in the **Online Supplementary Document**).

**Figure 3:**
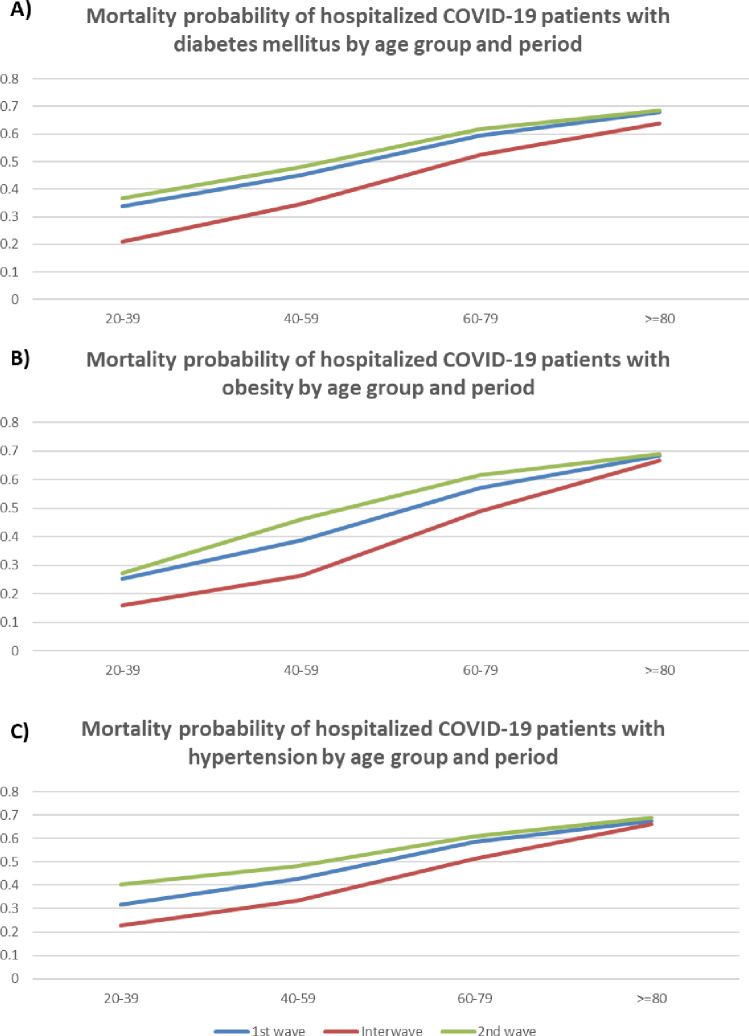
A) Mortality probability of hospitalized COVID-19 patients with diabetes mellitus by age group and period, B) Mortality probability of hospitalized COVID-19 patients with diabetes mellitus by age group and period, and C) Mortality probability of hospitalized COVID-19 patients with hypertension by age group and period using Bayes probability test.

The probability of dying among individuals suffering from DM, obesity, or AHP and COVID-19 in the general population showed a systematic reduction in all age groups, (Tables S4-S16 in the **Online Supplementary Document**). In contrast, the probability of dying for hospitalized individuals with DM (**Figure 3** and Table S13 in the **Online Supplementary Document**), who suffered from obesity (**Figure 3** and Table S15 in the **Online Supplementary Document**), or who suffered from AHP (**Figure 3** and Table S14 in the **Online Supplementary Document**) increased in all age groups systematically during the second wave.

### Differences in lethality among hospitalized patients with COVID-19 during waves and the interwave period

We used the Fisher exact test to identify whether individual hospitalized populations exhibited changes in lethality as the pandemic evolved. The first comparison contrasted lethality in the IP with that of the two waves. **Table 3** shows changes in CFR for hospitalized patients with the subpopulations found significant using Fisher exact test (FDR<0.05). We observed increases in lethality across all age groups. In each age group, the subpopulation ranked first, in terms of FDR, which was the one composed of patients without comorbidities. However, in terms of the magnitude of the lethality increase (in terms of the difference in CFR), each age group behaved differently. In the case of the 20 to 39 group, the largest increase was found in the subpopulation with DM (Table S16 in the **Online Supplementary Document**) and AHP(Table S17 in the **Online Supplementary Document**); in the 40 to 59 group, the subpopulations with DM, AHP (Table S18 in the **Online Supplementary Document**), and obesity showed the largest increase; in the 60 to 79 group the subpopulation with obesity registered the highest increase; finally, in patients aged 80 and over, the largest increase in lethality was in the subpopulation without recorded comorbidities. When comparing lethality between the FW and the SW, only the subpopulation of patients aged 40 to 59 without comorbidities showed a significant (FDR = 4.46E-02) yet small (change in CFR = 2.09) increase. Attributable and relative risks for each comorbidity are shown in **Table 4**.

**Table 3:** Lethality comparison between waves and the interwave period among hospitalized patients with COVID-19

Age group	Comorbidities	CFR-interwave	CFR-waves	Difference-CFR	Fisher exact test FDR*
20,39	None	7.91	11.65	3.74	1.42x10^-5^
20,39	Obes	13.00	18.21	5.21	1.50x10^-2^
20,39	DM, AHP	16.07	32.55	16.48	3.37x10^-2^
20,39	DM, Obes	13.46	28.76	15.30	4.48x10^-2^
40,59	None	20.37	29.72	9.34	2.29x10^-27^
40,59	Obes	22.35	33.79	11.45	1.30x10^-11^
40,59	DM, AHP, Obes	27.72	42.02	14.30	1.65x10^-6^
40,59	AHP	25.57	35.33	9.76	2.35x10^-6^
40,59	DM, AHP	32.86	42.33	9.47	2.35x10^-6^
40,59	AHP, Obes	26.04	37.69	11.64	5.00x10^-5^
40,59	DM	30.10	38.08	7.98	6.81x10^-5^
40,59	DM, Obes	26.72	38.52	11.80	1.20x10^-3^
40,59	Other	24.36	29.15	4.79	2.51x10^-2^
60,79	None	40.85	49.89	9.04	8.82x10^-16^
60,79	DM	41.51	53.41	11.90	3.35x10^-10^
60,79	AHP	41.01	50.55	9.54	3.64x10^-9^
60,79	Obes	39.41	52.80	13.39	2.35x10^-6^
60,79	DM, AHP	46.79	53.64	6.84	2.35x10^-6^
60,79	Other	39.07	47.82	8.75	1.02x10^-4^
60,79	DM, AHP, Obes	45.36	54.21	8.85	4.10x10^-4^
60,79	DM, Obes	39.81	54.26	14.45	7.39x10^-4^
60,79	AHP, Obes	46.10	52.27	6.16	4.48x10^-2^
80+	None	50.47	59.02	8.55	8.76x10^-4^
80+	DM, AHP	51.75	58.02	6.27	4.48x10^-2^

DM – diabetes mellitus, AHP – arterial hypertension, Obes – obesity, CFR-wave – cases occurred in either peak, Difference-CFR – the difference between CFR-wave and CFR-interwave, CFR-interwave – cases occurred during the period interpeak, FDR – Fisher exact test

*Fisher exact test with Benjamini-Hochberg (BH) correction was used to estimate difference. FDR<0.05 – statistically significant value.

**Table 4:** Attributable and relative risks for each comorbidity among hospitalized patients with COVID-19

Health condition	P (POS)	Att. risk	Rel. risk	P (HOSP)	Att. risk	Rel. risk	P (DEATH)	Att. risk	Rel. risk
Chronic kidney disease	0.362	0.070	1.239	0.579	0.470	5.311	0.351	0.313	9.236
COPD	0.383	0.091	1.311	0.503	0.394	4.614	0.305	0.267	8.026
Cardiac disease	0.336	0.044	1.150	0.399	0.290	3.660	0.207	0.169	5.447
Diabetes mellitus	0.392	0.100	1.342	0.397	0.288	3.642	0.204	0.166	5.368
Immuno-suppression	0.291	-0.001	0.996	0.436	0.327	4.000	0.198	0.160	5.210
AHP	0.369	0.077	1.263	0.361	0.252	3.311	0.189	0.151	4.973
Obesity	0.384	0.092	1.315	0.237	0.128	2.174	0.105	0.067	2.763
Smoking	0.282	-0.010	0.965	0.160	0.051	1.467	0.073	0.035	1.921
Asthma	0.286	0.006	0.979	0.138	0.029	1.266	0.049	0.011	1.289
None	0.292	0.000	1.000	0.1090	0.000	1.000	0.038	0.000	1.000

AHP – arterial hypertension, COPD – chronic obstructive pulmonary disease, Att. risk – attributable risk, Rel. risk – relative risk

Finally, comparing the IP with the waves gave us a notion of changes in lethality associated with periods of increased transmission and demand for hospitalization. **Table 5** shows the results of this analysis. We observed statistically significant increases in several subpopulations (23 out of 48 evaluated), which again highlighted higher lethality in the period with increased hospital demand. Nevertheless, it should be noted that when comparing the global population of hospitalized and ambulatory COVID-19 patients, we do see some populations with significant decreases in lethality (Table S19 in the **Online Supplementary Document**), particularly in persons older than 60 years of age and younger populations with an increased risk of lethality.

**Table 5:** Lethality comparison between the interwave period and the second wave among hospitalized patients with COVID-19

Age group	Comorbidities	CFR-interwave	CFR-wave 2	Difference-CFR	Fisher exact test FDR*
20-39	None	7.95	18.75	10.80	4.22x10^-17^
20-39	AHP	15.12	38.62	23.51	2.03x10^-4^
20-39	Obes	13.64	23.10	9.46	5.17x10^-4^
20-39	Other	13.93	22.71	8.77	8.88x10^-3^
20-39	DM, Obes	16.67	39.19	22.52	3.36x10^-2^
40-59	None	22.77	37.86	15.09	1.03x10^-44^
40-59	Obes	23.95	42.30	18.35	1.36x10^-19^
40-59	AHP, Obes	27.11	48.45	21.34	2.97x10^-10^
40-59	DM, AHP, Obes	31.21	52.01	20.80	8.70x10^-9^
40-59	AHP	30.92	44.54	13.62	2.90x10^-7^
40-59	DM, Obes	27.63	49.22	21.59	3.12x10^-7^
40-59	DM	33.83	44.14	10.30	4.64x10^-5^
40-59	DM, AHP	39.90	50.26	10.36	8.06x10^-5^
40-59	Other	27.93	37.59	9.66	5.17x10^-4^
60-79	None	45.38	58.06	12.69	3.76x10^-23^
60-79	AHP	46.37	59.92	13.55	1.13x10^-12^
60-79	DM	48.04	61.32	13.27	4.61x10^-9^
60-79	DM, AHP, Obes	50.75	65.06	14.31	4.64x10^-7^
60-79	Obes	45.38	60.55	15.17	2.76x10^-6^
60-79	Other	46.57	59.55	12.98	5.12x10^-6^
60-79	DM, AHP	55.46	61.41	5.95	7.19x10^-4^
60-79	AHP, Obes	49.30	59.39	10.09	2.62x10^-3^
80+	None	58.04	68.08	10.04	5.58x10^-4^

DM – diabetes mellitus, AHP – hypertension, Obes – obesity, CFR-wave – cases occurred in either peak, difference-CFR – the difference between CFR-wave and CFR-interwave, CFR-interwave – cases occurred during the interpeak period, FDR – Fisher exact test

*Fisher exact test with BH correction was used to estimate significance. FDR<0.05 – statistically significant value.

### Correlation of hospital occupancy with hospital mortality

**Figure 4:**
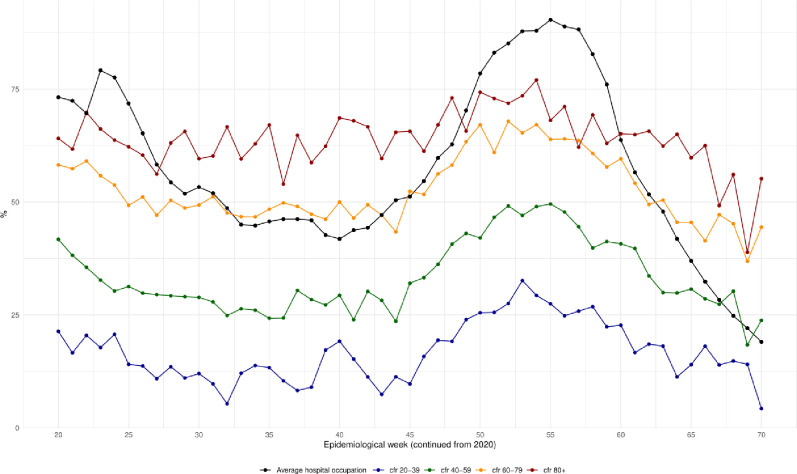
Pearson correlation between weekly case fatality rate (CFR) for hospitalized patients and hospital occupancy. Lethality increases during periods of higher transmission and demand for hospitalization. It was observed that lethality across all age groups decreased as hospital occupation improved at the end of the first wave and increased again during the second wave.

Considering that lethality increases during periods of higher transmission and demand for hospitalization, we calculated the Pearson correlation between weekly CFR for hospitalized patients and hospital occupancy. **Figure 4** shows the time series for CFR and hospital occupancy. We observed that lethality across all age groups decreased as hospital occupation decreased at the end of the FW, but then increased again during the SW. **Table 6** shows the correlation between weekly hospital occupation and CFR per age group, the correlation values were high in all age groups.

**Table 6:** Correlation between weekly hospital occupation and case fatality rate per age group

Age group	Correlation
20-39	0.76
40-59	0.84
60-79	0.90
80+	0.65

## DISCUSSION

The data from this registry-based study come from a population of more than two million people analyzed and reveal that even though a considerable increase was observed in the number of cases of infection (+472%), in the number of hospitalized subjects (+85.9%), and the number of hospitalized subjects and deaths (+102.4%) in all age groups between both waves, the probability of death diminished in the general population during the period studied, which is most probably due to the greater number of subjects studied after the first wave. Nevertheless, when analyzing only those hospitalized individuals, with or without comorbidities, the CFR was high (50.2%). The probability of death increased considerably in all age groups, and this increase was more noticeable in individuals with previously identified comorbidities (DM, AHP, or obesity). After concluding this analysis, we were struck by the increased probability of death in the groups of individuals without comorbidities, particularly in the groups aged 20 to 39 years and 40 to 59 years during wave episodes. This indicated to us that the youngest individuals had a very significant risk of death due to the severity of the disease which had led to their hospitalization. This result forces us to maintain the protective measures to avoid infection by the SARS-Cov-2 virus in the entire population, to intensify the vaccination program in young adults and adolescents and improve the medical care of those individuals requiring hospitalization, but with emphasis on the groups that we have identified as having significant risk.

Staerk et al. [21] described how effective IFRs (infection fatality rates) were estimated to vary over time as the age distributions of confirmed cases and estimated infections were changing during the pandemic. Alimohamadi et al. reported an interesting global analysis [22] of the CFR of COVID-19: the overall pooled CFR of COVID-19 was 10.0% (95% CI = 8.0-11.0). The pooled CFR of COVID-19 among the general population was 1.0% (95% CI = 1.0-3.0); while in hospitalized patients it was 13.0% (95% CI = 9.0-17.0). The pooled CFR in patients admitted into intensive care units was 37.0% (95% CI = 24.0-51.0), and in patients aged over 50, it was 19.0% (95% CI = 13.0-24.0). In our study, we observed a very high CFR in hospitalized COVID-19 patients (50.2%) because of the severity of the infection and its complications, although the high CFR can also be explained by the harmful effect of the saturation of hospital services occurring during the two waves (as can be seen in **Figure 2**) especially those between 20 and 59 years of age. Our group has previously reported that 45% of patients who did not survive in a tertiary care hospital were not admitted to the ICU due to the lack of availability of ICU beds [23]. Therefore, the mortality rate over time was mainly due to the availability of ICU beds, indirectly suggesting that overcrowding was one of the main contributing factors to hospital mortality. Above all, in this study, the risk observed in people of these age groups (under 60 years) without associated comorbidities is striking.

The risk of SARS-COV-2 infection associated with being over 60 was reported early during the pandemic [24]. Additionally, in both China and the USA, DM, AHP, and obesity were reported as comorbidities that increase the risk of severe disease, hospitalization, and death [25]. Also early on, a respiratory-onset viral infection was identified as generating progressive multi-organ disease leading initially to respiratory failure and later to multi-organ failure [26]. All the above is related to an inflammatory phenomenon called a cytokine storm [27], which causes extensive tissue damage with acute functional deterioration and specific organic sequelae in certain groups of patients [28].

Early on, in Mexico and Latin America, several groups highlighted the rapid progression of patients with COVID-19 [12,29-32] in coexistence with comorbidities such as DM, AHP, and obesity, as being a worrying situation due to the high prevalence of overweight/obesity, metabolic syndrome and DM in the Mexican population as per information collected by the latest ENSANUT [8]. Then again, we have observed that a good proportion of COVID-19 patients are people in their fourth and fifth decades of life, younger than the patients in the USA reports. Notably, we observed a great increase in the number of hospitalizations between the two waves, indicative of the severity of the pandemic in the CDMX and evidence of the intense transmission that occurred in that period, there being a notable predominance of a variant designated B.1.1.519. We observed an overlap of the SW with the emergence of variant B.1.1.519, which represented 74.3% of the sequences generated in CDMX (2296/3092) from November 2020 to May 2021, distributed evenly across CDMX [15].

We observed that across all age groups, there was an increase in hospitalizations during the SW, compared with the FW. The relative growth in the number of hospitalizations as a function of age ranged from a +36.2% increase in the 20-39 age group up to a +171.37% increase in the 80+ group, as shown in **Figure 1**. This is evidence that all age groups contributed to the greater pressure on hospital occupation during the SW, with the most notably increasing demand being in the older-aged groups. Similarly, we observed an increase in the number of deaths and an increase in the probability of death in all age groups with or without comorbidities. We observed that the probability of dying among individuals with DM, obesity, or AHP and COVID-19 in the general population was initially reduced in all age groups because of the +800% increase in the number of subjects tested during the study when comparing FW and SW. However, the probability of death in individuals with DM, AHP, or obesity who were hospitalized increased during the SW in all age groups, confirming the associated risk observed among the population.

As a result of this careful analysis, we have been able to identify the increased risk of complications and death, between the FW and SW, in all groups of hospitalized patients without comorbidities, from 3.74% in the group aged 20 to 40 to 9.34% in the group of patients aged 40 to 60, which reveals the risk to this population, primarily considered to be healthy. In the healthy population, the appearance of the multisystem inflammatory syndrome has been widely described, especially in children [33], although at the beginning of the pandemic, it was confused with Kawasaki syndrome. A similar pattern has been described in adults from various regions of the world, in whom a picture of shock rather than respiratory failure [33,34] has been observed, associated with a high risk of death. In such patients, the PCR tests may be negative because they can manifest during the acute phase of the disease or as a postinfectious condition. This clinical scenario has been observed in Mexican children and recently reported [35]. In this report, 62% of the studied population did not report previous diseases and it is conceivable that a fraction of this population had presented with this multisystem inflammatory syndrome, however, we recognize that we cannot affirm that this would have been the diagnosis. Therefore, more research is required to ascertain if this condition exists in the Mexican adult population.

Attention has recently been drawn to the appearance of severe COVID-19 in groups of people without comorbidities. A study recently published by ISARIC WHO Clinical Characterization Protocol (UK) draws attention to the appearance of serious disease in this group of people and their need for hospitalization. In consequence, they have pointed out that it is helpful to look for other outcomes such as disability or chronic damage that may appear in the members of this group who survive but may develop complications in the medium and long term [7]. Recently, loci and genes associated with susceptibility to COVID-19 or severe COVID-19 have been identified using comprehensive GWAS (Genome-wide association studies) or analysis of genome, exome, or candidate genes [36-43].

Based on our results, the results of the study carried out by our group in the tertiary care hospital [23] and the results of other recently published studies [30], we consider that it is necessary to carry out the following measures to avoid an increase in the rates of lethality in hospitalized patients. The first measure is, without a doubt, to intensify public health measures shown to decrease transmission (use of face masks, social distancing, and continuous application of hygiene measures). The second is the intensification of vaccination among different age groups progressively until the entire population is covered. In this way, we can significantly reduce the number of cases of hospitalization, episodes of aggravation of the disease, transferences to intensive care units, and the need for endotracheal intubation, as well as avoid cases of disability and death (the data for CDMX are currently under analysis). The third measure is the promotion of early care to recognize patients with risk comorbidities or those individuals with clinical manifestations or alterations in laboratory tests, indicative of severity or progressive deterioration [44].

As regards the limitations and strengths of this study, its main limitation was that the recruitment method of the persons infected by SARS-CoV-2 was modified from symptomatic during the first wave to both asymptomatic and symptomatic in the other two periods, in addition to the modification of the diagnostic procedure from tPCR to antigen detection for the screening method. This condition prevented an adequate calculation of the infection fatality rate since the inclusion of symptomatic individuals predominated in the screening system. Another significant limitation was being unable to follow up on all the contacts of at least hospitalized patients to gain a better understanding of the severity of transmission. Furthermore, there were problems registering deaths and difficulties completing the registration, especially during the first wave. Therefore, for patients who died without a PCR-SARS-CoV-2 test, a “probable COVID-19” code was added to include those patients without a confirmatory test. On the other hand, although this was not designed as a population-based or cohort study, the study’s main strength remains in its inclusion and consideration of all hospitalized patients, exploring their outcomes, and carrying out the entire probabilistic and Bayesian analysis. Although the SISVER database might include some “measurement uncertainty”, the Bayesian model is not affected by this kind of “uncertainty” since the potential biases were incorporated into the priors from the start. Thus, the resulting analysis model was in some sense “strengthened” by this strategy.

## CONCLUSIONS

This observational study revealed a considerable increase in the number of cases (+472%) of infection plus an +85.9% increase in hospitalized cases in all age groups between the first and second waves. Additionally, 12.8% of the infected persons were hospitalized because of severe COVID-19, with a high mortality rate among the hospitalized patients (>50%). Case Fatality Rates among hospitalized patients were higher during the peaks than during the interwave period. A higher probability of death was observed among hospitalized patients who suffered from DM, AHP or obesity. This probability increased during periods of higher hospital demand. It should be noted that this increase in the risk of death was observed in all age groups, regardless of the presence or absence of comorbidities.

## Additional material


Online Supplementary Document
