# Supplementary material for: Probability of hospitalisation and death among COVID-19 patients with comorbidity during outbreaks occurring in Mexico City
Source: J Glob Health. 2022 Nov 8;12:05038. doi: 10.7189/jogh.12.05038 (PMC9639670; doi:10.7189/jogh.12.05038)
Supplement: Online Supplementary Document [file jogh-12-05038-s001.pdf]

ONLINE SUPPLEMENTARY DOCUMENT

Title: PROBABILITY OF HOSPITALIZATION AND DEATH AMONG COVID-19 PATIENTS WITH COMORBIDITY DURING OUTBREAKS OCCURRING IN MEXICO CITY.

Authors: José Sifuentes-Osornio[1]*, Ofelia Angulo-Guerrero[2]*, Guillermo De-Anda-Jáuregui[3,4]*, Juan L. Díaz-De-León-Santiago[2]*, Enrique Hernández-Lemus[3,4], Héctor Benítez-Pérez[7], Luis A. Herrera[3,6], Oliva López-Arellano[2,] Arturo Revuelta-Herrera[2], Ana R. Rosales-Tapia[5], Rosaura Ruiz-Gutiérrez[2,8], Manuel Suárez-Lastra[5], David Kershenobich[1].

Table S1. Contingency table used to perform 1) Comparison between hospitalized patients during either of the two waves and in the interwave period and 2) Comparison between hospitalized patients in the first peak and the second peak.

	Death	No death
Period A	The death count in Period A	Survivor (cases that did not end with death) count in Period A
Period B	The death count in Period B	Survivor (cases that did not end with death) count in Period B

Table S2. Global lethality probability of COVID-19 patients by age group and period.

Age group (years)	*1st wave*	*Interwave Period*	*2nd wave*
20-39	0.024	0.005	0.005
40-59	0.122	0.042	0.039
60-79	0.359	0.193	0.167
≥ 80	0.498	0.391	0.328

Bayes probability test.

Table S3: Mortality probability of hospitalized COVID-19 patients by age group and period.

Age group (years)	*1st wave*	*Interwave period*	*2nd wave*
20-39	0.185	0.116	0.228
40-59	0.368	0.274	0.418
60-79	0.577	0.486	0.560

≥80	0.673	0.630	0.682

Bayes probability test

Table S4. Frequencies of people with diabetes mellitus, positive for SARS-CoV-2 infection, hospitalized and deceased during the first wave (15 weeks, between March 23 to July 12, 2020).

Age group (years)	People with diabetes	People with SARS-CoV-2 infection	People with COVID-19 and hospitalized	Deceased people with COVID-19	People with COVID-19, hospitalized and deceased
20-39	1970	940	314	123	106
40-59	11812	6017	2754	1399	1245
60-79	9225	5299	3321	2202	1980
>80	1316	703	490	374	332

Table S5. Frequencies of people with diabetes mellitus, positive for SARS-CoV-2 infection, hospitalized and deceased during the interwave period (14 weeks, July 13 to October 25, 2020).

Age group (years)	People with diabetes	People with SARS-CoV-2 infection	People with COVID-19 and hospitalized	Deceased people with COVID-19	People with COVID-19, hospitalized and deceased
20-39	2661	1001	166	37	35
40-59	16669	6607	1723	643	598
60-79	13409	5790	2620	1473	1373
>80	1615	751	475	328	304

Table S6. Frequencies of people with diabetes mellitus, positive for SARS-CoV-infection, hospitalized and deceased during the second wave (25 weeks, between 26 October 2020 to 29 March 2021).

Age group (years)	People with diabetes	People with SARS-CoV-2 infection	People with COVID-19 and hospitalized	Deceased people with COVID-19	People with COVID-19, hospitalized and deceased
20-40	10850	3407	374	145	137

40-60	67455	22818	3993	2079	1920
60-80	52223	20483	6750	4513	4178
>80	5442	2458	1226	903	839

Table S7. Frequencies of people with hypertension, positive for SARS-CoV-2 infection, hospitalized and deceased during the first wave (15 weeks, March 23 to July 12, 2020).

Age group (years)	People with hypertension	People with SARS-CoV-2 infection	People with COVID-19 and hospitalized	Deceased people with COVID-19	People with COVID-19, hospitalized and deceased
20-40	2691	1124	297	115	94
40-60	13836	6548	2627	1269	1125
60-80	11714	6548	3833	2515	2248
>80	2293	1218	817	623	551

Table S8. Frequencies of people with hypertension, positive for SARS-CoV-2 infection, hospitalized and deceased during the interwave period (14 weeks, July 13 to October 25, 2020).

Age group (years)	People with hypertension	People with SARS-CoV-2 infection	People with COVID-19 and hospitalized	Deceased people with COVID-19	People with COVID-19, hospitalized and deceased
20-40	3889	1313	203	50	46
40-60	21018	7772	1750	627	584
60-80	18327	7458	3074	1696	1576
>80	2913	1316	764	543	504

Table S9. Frequencies of people with hypertension, positive for SARS-CoV-2 infection, hospitalized and deceased during the second wave (25 weeks, between Oct 26, 2020, to March 29, 2021).

Age group (years)	People with hypertension	People with SARS-CoV-2 infection	People with COVID-19 and hospitalized	Deceased people with COVID-19	People with COVID-19, hospitalized and deceased
20-40	16464	4815	425	181	171
40-60	86466	27486	4141	2185	1999
60-80	72959	27040	8257	5446	5049
>80	10082	4392	2091	1547	1437

Table S10. Frequencies of people with obesity, positive for SARS-CoV-2 infection, hospitalized and deceased during the first wave (15 weeks, March 23 to July 12, 2020).

Age group (years)	People with obesity	People with SARS-CoV-2 infection	People with COVID-19 and hospitalized	Deceased people with COVID-19	People with COVID-19, hospitalized and deceased
20-40	11062	4698	773	230	195
40-60	16118	7834	2600	1170	1007
60-80	5049	2984	1673	1094	958
>80	448	247	152	122	104

Table S11. Frequencies of people with obesity, positive for SARS-CoV-2 infection, hospitalized and deceased during the interwave period (15 weeks, July 13 to October 25, 2020).

Age group (years)	People with obesity	People with SARS-CoV-2 infection	People with COVID-19 and hospitalized	Deceased people with COVID-19	People with COVID-19, hospitalized and deceased
20-40	15966	5788	473	88	76
40-60	20720	8271	1717	497	454

60-80	6635	3056	1370	722	670
>80	506	240	153	110	102

Table S12. Frequencies of people with obesity, positive for SARS-CoV-2 infection, hospitalized and deceased during the second wave (25 weeks, between October 26 to March 29, 2021).

Age group (years)	People with obesity	People with SARS-CoV-2 infection	People with COVID-19 and hospitalized	Deceased people with COVID-19	People with COVID-19, hospitalized and deceased
20-40	55280	16457	1004	299	273
40-60	65931	22055	3713	1851	1714
60-80	19573	8458	3436	2258	2120
>80	1481	735	436	321	300

Table S13. Probability of death of COVID-19 patients with diabetes mellitus according to age groups and pandemic waves.

Age group (years)	1st wave	Interwave	2nd wave
20-39	0.130	0.036	0.0425
40-59	0.232	0.097	0.091
60-79	0.415	0.254	0.220
≥80	0.532	0.436	0.367

Table S14. Probability of death of COVID-19 patients with hypertension according to age groups and pandemic waves.

Age group (years)	1st wave	Interwave	2nd wave
20-39	0.102	0.038	0.037
40-59	0.193	0.080	0.079
60-79	0.384	0.227	0.201
≥80	0.511	0.412	0.352

Table S15. Probability of death of COVID-19 patients with obesity according to age groups and pandemic waves.

Age group (years)	1st wave	Interwave	2nd wave

20-39	0.048	0.015	0.018
40-59	0.149	0.060	0.083
60-79	0.366	0.236	0.266
≥80	0.493	0.458	0.436

Table S16. Mortality probability of hospitalized COVID-19 patients with diabetes mellitus by age group and period.

Age group (years)	*1st wave*	*Interwave period*	*2nd wave*
20-39	0.338	0.210	0.366
40-59	0.452	0.347	0.481
60-79	0.596	0.524	0.619
≥80	0.678	0.640	0.684

Bayes probability test.

Table S17: Mortality probability of hospitalized COVID-19 patients with hypertension by age group and period.

Age group (years)	*1st wave*	*Interwave period*	*2nd wave*
20-39	0.316	0.227	0.402
40-59	0.428	0.334	0.483
60-79	0.586	0.513	0.611
≥80	0.674	0.660	0.687

Bayes probability test.

Table S18: Mortality probability of hospitalized COVID-19 patients with obesity by age group and period.

Age group (years)	*1st wave*	*Interwave period*	*2nd wave*
20-39	0.252	0.161	0.272
40-59	0.387	0.264	0.462
60-79	0.572	0.489	0.617
≥80	0.684	0.667	0.688

Bayes probability test.

Table S19. Comparison of lethality (in ambulatory and hospitalized patients) between interwave period and the second wave.

Age group (years)	Comorbidities	CFR_interwave	CFR_2nd wave	Difference-CFR	FDR
[60,79)	DM; AHP	27.72	22.06	-5.66	9.72E-08
[60,79)	None	15.25	12.78	-2.46	2.24E-06

[40,59)	Obes	4.48	6.10	1.62	2.26E-04
[40,59)	AHP; Obes	6.43	9.82	3.40	7.25E-04
[40,59)	DM; AHP; Obes	11.45	16.49	5.03	3.53E-03
[80,100)	DM; AHP	44.61	36.19	-8.41	8.85E-03
[60,79)	Obes	18.61	23.20	4.58	2.20E-02
[80,100)	none	34.56	29.18	-5.38	3.09E-02
[40,59)	DM; AHP	13.63	11.28	-2.35	3.38E-02
[60,79)	DM	20.11	17.48	-2.62	4.13E-02

DM: diabetes mellitus; AHP: hypertension arterial; Obes: obesity; CFR-wave: cases occurred in either peak; difference-CFR: the difference between CFR-wave and CFR-interwave; CFR-interwave: cases occurred during the interpeak period; statistical significant value: FDR<0.05. Periods: interwave: Epidemiological weeks 29-43 of 2020 (14 weeks, July 13 to October 25). Second wave: Epidemiological weeks 44/2020 -13/2021 (25 weeks, October 26 to March 29). Fisher exact test with BH correction used to estimate significance. Positive values in Difference-CFR represent **increases** in lethality. Negative values in Difference-CFR **represent decreases in** lethality.
